# Erosive Pustular Dermatosis of the Scalp in a Patient With Vitiligo Successfully Treated With Topical Antibiotics

**DOI:** 10.7759/cureus.45913

**Published:** 2023-09-25

**Authors:** Shamma Khamis Almheiri, Esmaeel Alsayed Mohamed Almrzooqi

**Affiliations:** 1 Dermatology, Dubai Academic Health Corporation, Dubai, ARE

**Keywords:** dermatology, geriatrics, topical antibiotics, sun-damaged skin, actinic keratosis, vitiligo, erosive pustular dermatosis of the scalp

## Abstract

Erosive pustular dermatosis of the scalp is a rare inflammatory skin disorder that most commonly affects the elderly with androgenic alopecia. It is characterized by painless crusted erosions that usually appear following a traumatic event. It has been associated with several autoimmune diseases and commonly responds to high-potency topical steroids.This report presents a rare case of erosive pustular dermatosis of the scalp in an 84-year-old man who presented to our clinic.

## Introduction

Erosive pustular dermatosis is an uncommon chronic inflammatory skin disorder [1]. The lesions typically present on the scalp of elderly patients with sun-damaged skin and hair loss [1-4]. It develops over months to years, presents as asymptomatic erosions covered with a yellow-crusted discharge, and is frequently associated with pustules, skin atrophy, and scarring alopecia [1-4]. There are several precipitating factors for the development of erosive pustular dermatosis, all of which ultimately lead to skin injury [1,2,5]. An association with multiple autoimmune conditions has been reported [1-3].

The mainstay treatment for erosive pustular dermatosis is high-potency topical steroids [1,2,4]. This report describes a rare case of erosive pustular dermatosis of the scalp developing in a patient with vitiligo that healed following the use of topical antibiotics.

## Case presentation

An 83-year-old male presented to our clinic complaining of a non-healing ulcer on his scalp. The ulcer appeared on his scalp four months ago and developed at the site of an existing lesion of actinic keratosis following a cryotherapy session. The ulcer is painless and non-itchy. It has been increasing in size over the past four months and is associated with a yellowish foul-smelling discharge. The patient did not relate the progression of the ulcer to sun exposure. He never used sunscreen or clothes to cover his scalp. He does not have any other ulcers on his skin and has never had them in the past. 

The patient is a known case of ischemic heart disease, depression, melanoma, and vitiligo. In 1997, he underwent coronary artery bypass grafting, followed by an angioplasty in 2019. The melanoma appeared on his forehead four years ago and was treated with surgical excision. He developed vitiligo 20 years ago but has never undergone any treatment for it, and he is not keen on starting any treatments. He currently takes the following medications daily: 75 mg of aspirin, 35 mg of trimetazidine, 10 mg of rosuvastatin, and 25 mg of quetiapine. He has no known allergies and no significant social or travel history.

**Figure 1:**
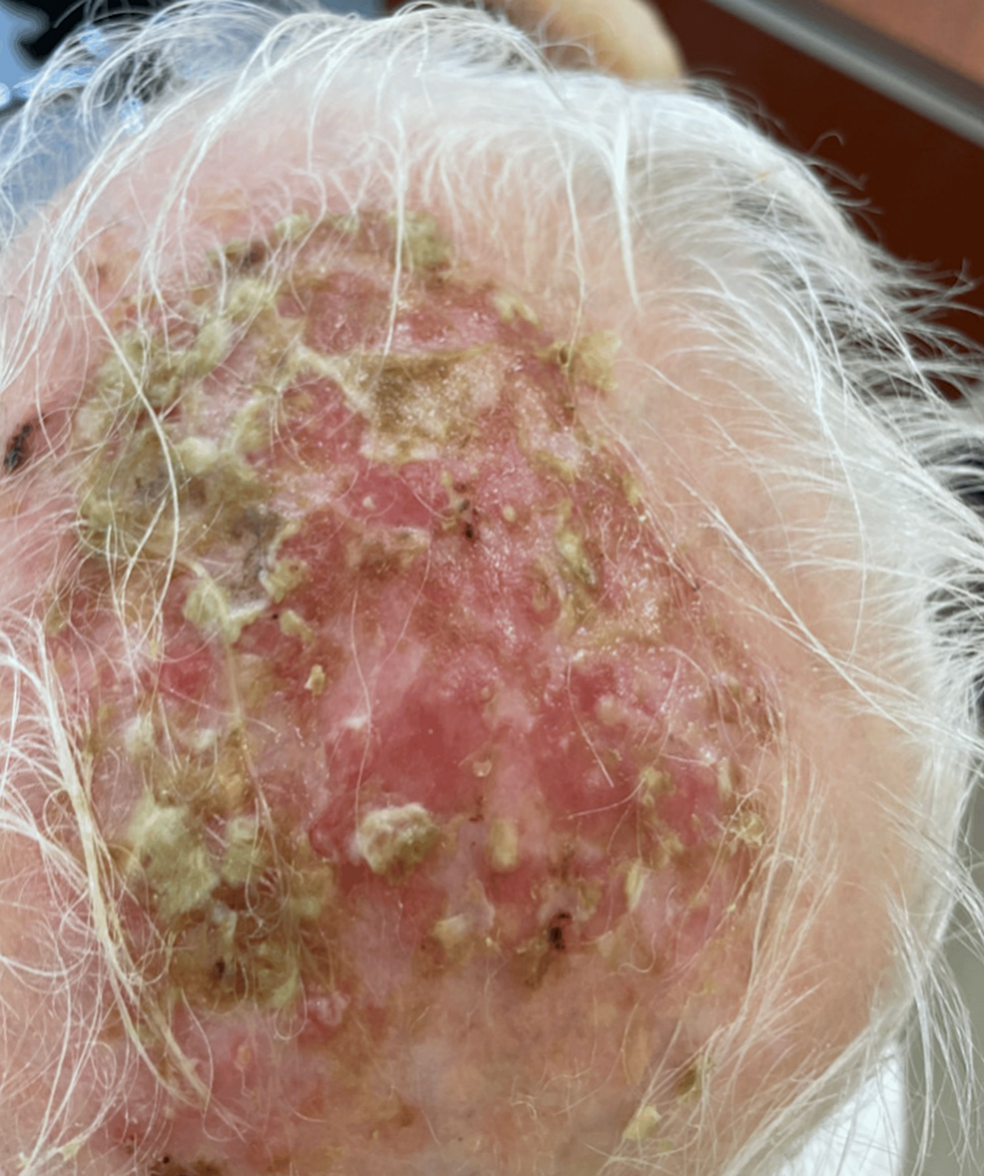
The condition of the scalp at presentation. A 6 cm by 8 cm erosion is observed on the vertex of the scalp, characterized by an erythematous floor with an overlying yellow-green foul-smelling exudate in the area of androgenic alopecia.

On physical examination, the patient was vitally stable. Examination of the scalp revealed a 6 cm by 8 cm erosion on the vertex of the scalp, characterized by an erythematous floor with an overlying yellow-green foul-smelling exudate in the area of androgenic alopecia. Other skin findings included an erythematous patch with gritty scales on the right side of the forehead, a hypopigmented macule on the left side of the forehead (previous site of melanoma excision), and extensive vitiligo of the skin (universal type). Systemic examination and examination of the nails and mucosae revealed no abnormality (Figure 1).

**Figure 2:**
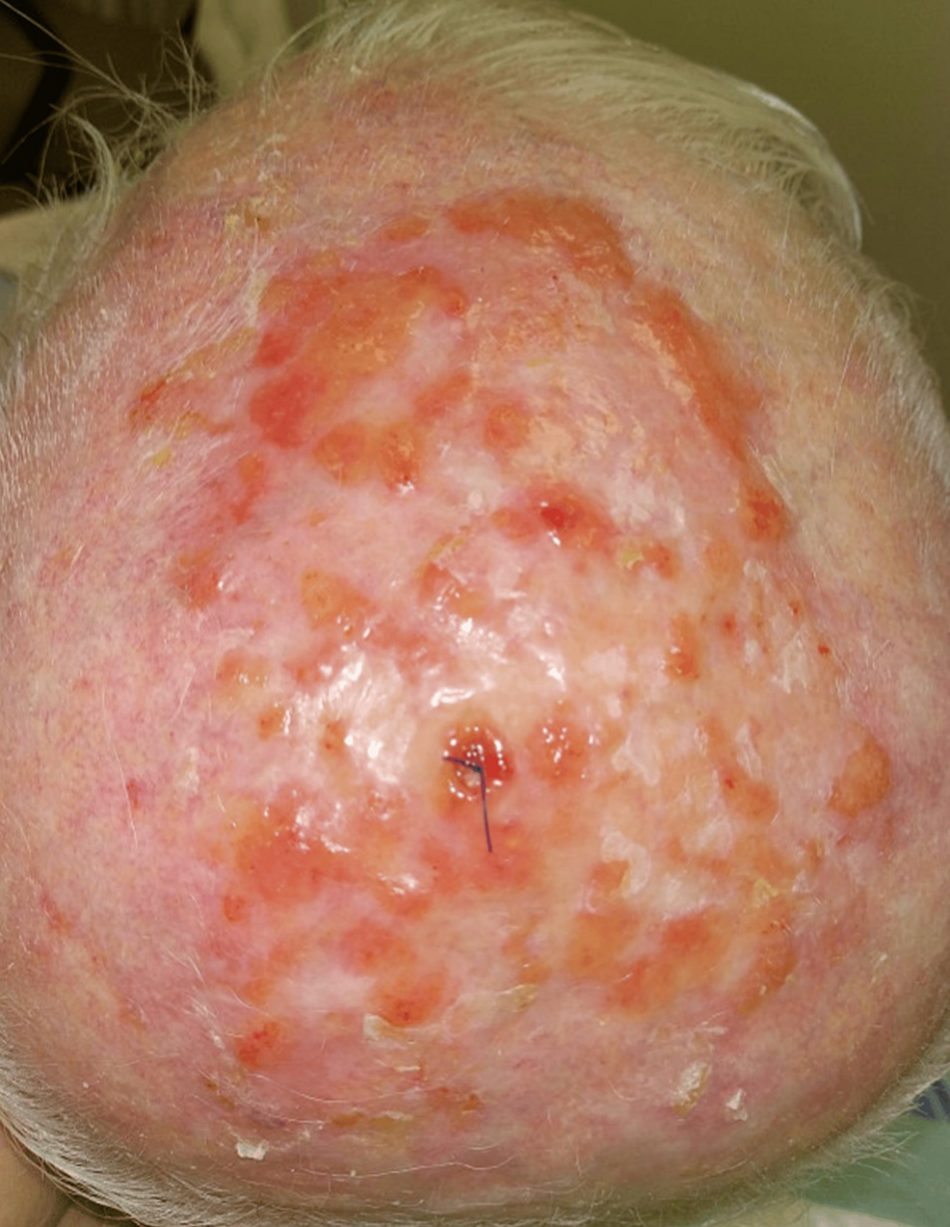
The condition of the scalp after cleaning. The scalp was cleaned with Betadine and Dermacyn solutions. All crusts were removed.

A wound culture and a skin biopsy were collected from the skin lesion. In addition, in view of the exudate seen on the lesion, the patient was prescribed two topical ointments to apply on the affected area (bismuth subgallate ointment, four times a day for 14 days, and mupirocin 2% ointment, two times a day for seven days). The patient was referred to the wound clinic, where the scalp was cleaned with Betadine and Dermacyn solutions (Figure 2). 

**Figure 3:**
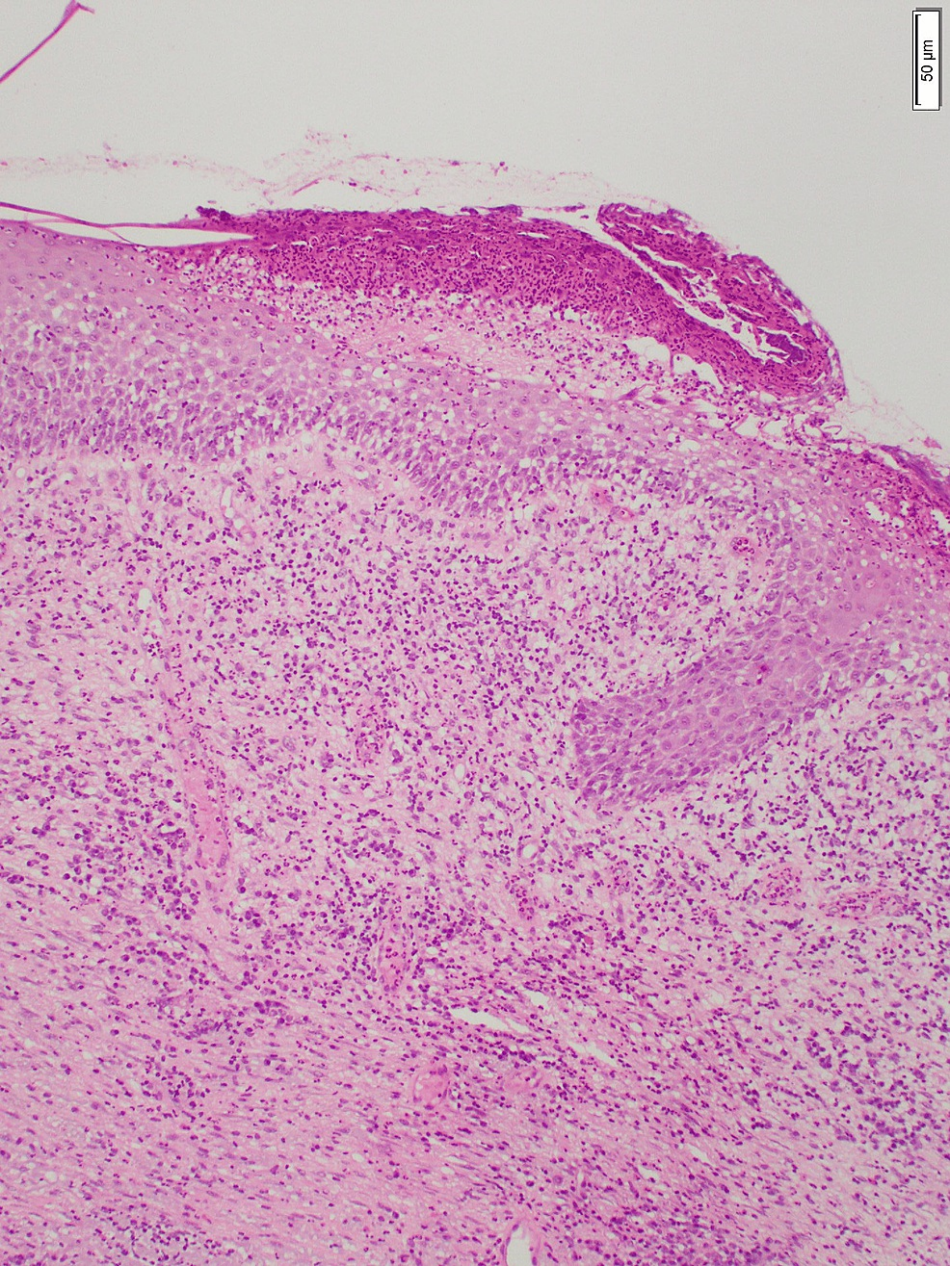
Skin biopsy findings. The histology slide shows non-specific findings, including ulceration, acute inflammation involving the superficial and deep dermis, solar elastosis, and no evidence of malignancy in the biopsy material.

The skin biopsy showed ulceration, acute inflammation involving the superficial and deep dermis, solar elastosis, and no evidence of malignancy (Figure 3). The wound culture showed a heavy growth of *Klebsiella oxytoca* and *Pseudomonas aeruginosa,* and both organisms were susceptible to all antibiotics. The patient was diagnosed with erosive pustular dermatosis of the scalp with secondary bacterial colonization. 

**Figure 4:**
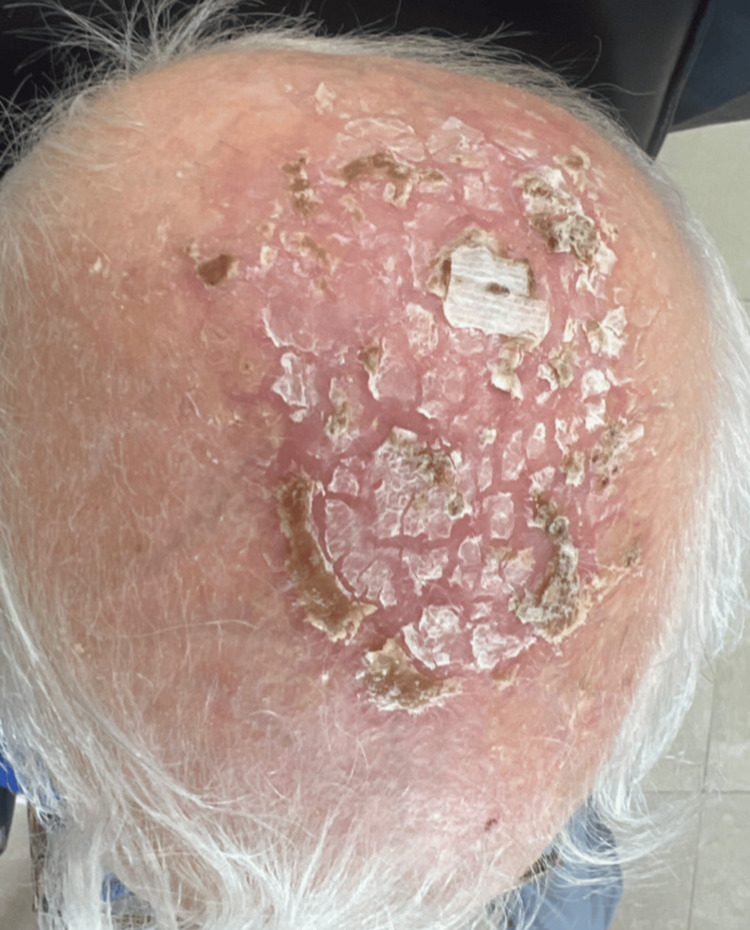
Two weeks of follow-up. The figure shows the condition of the scalp after two weeks of presentation. The erosions and ulcers healed completely, and no new lesions formed on the scalp.

The patient attended the clinic two weeks later for follow-up. The scalp showed marked improvement and no new lesions (Figure 4). The patient was instructed to use sunscreen regularly and wear protective clothing. 

**Figure 5:**
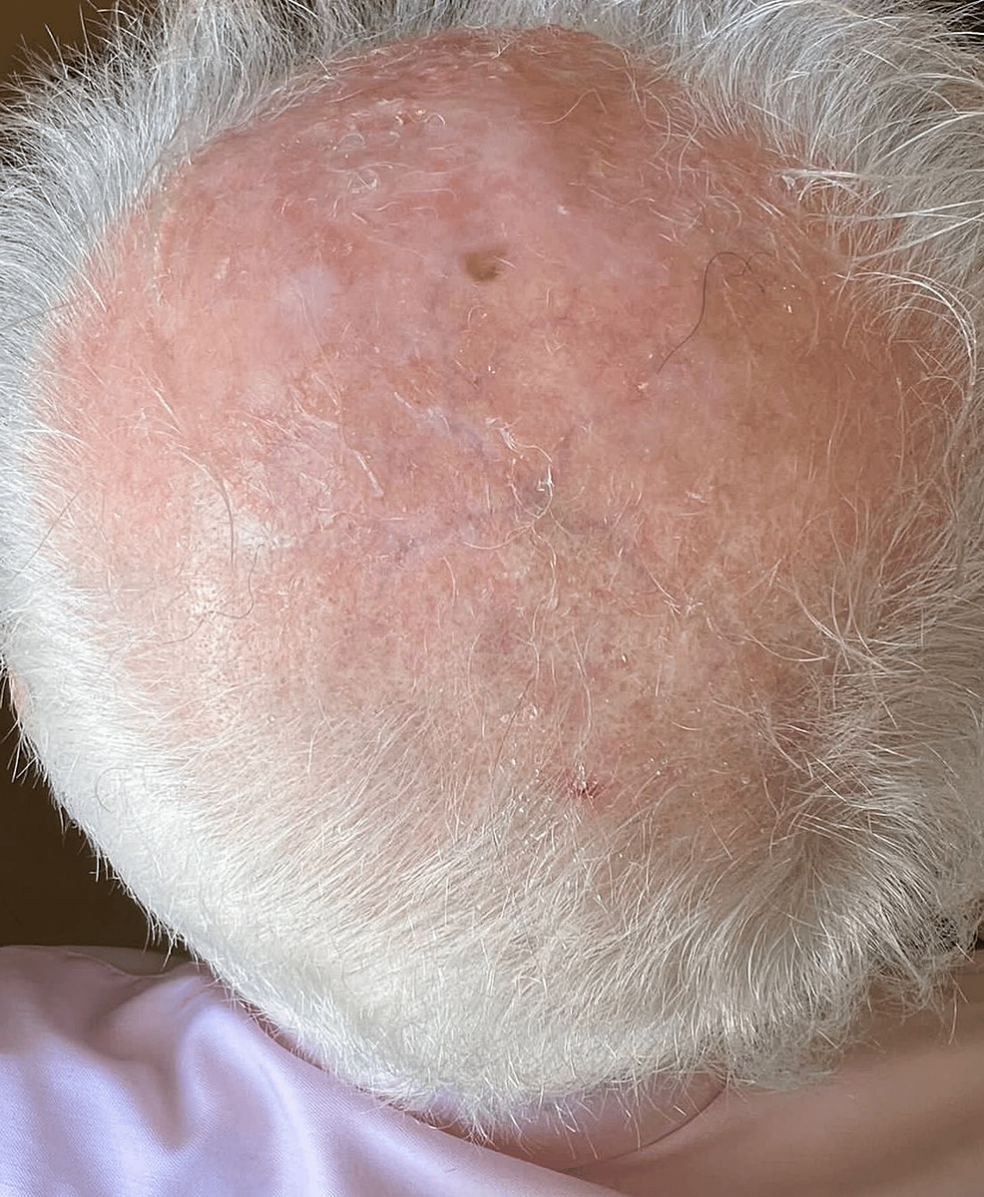
The condition of the scalp four months later. The figure presents the condition of the scalp four months after treatment. The scalp is normal and shows no new lesions.

One month later, the patient presented for follow-up. The scalp showed marked improvement and no new lesions. Treatment for the actinic keratosis on the forehead was resumed using cryotherapy and imiquimod 5% cream. After four months, the patient’s scalp returned to normal (Figure 5). 

## Discussion

Erosive pustular dermatosis is a rare skin disorder [1]. It was first described in 1977 by Dr. Burton [2]. Predisposing factors include sun damage and epidermal atrophy [1]. It typically develops following an insult to the skin. Some examples of skin insults include burns, physical injury, cryotherapy, topical treatments such as 5-fluorouracil, imiquimod, minoxidil, and tretinoin, and systemic medications such as afatinib, gefitinib, and erlotinib [1,2,5,6]. It can be associated with autoimmune disorders, including Hashimoto’s thyroiditis, autoimmune hepatitis, Takayasu’s arteritis, rheumatoid arthritis, and myasthenia gravis [1-3]. To the best of our knowledge, this is the only case to be reported in a patient with vitiligo. 

There is no gold standard treatment for erosive pustular dermatosis; however, high-potency topical steroids have been shown to have the best results [1,2,4,5]. Topical steroids lead to significant improvement within one to two months of use. However, it is well known that long-term use of topical steroids leads to skin atrophy, which leaves the patient with weaker skin and serves as a predisposing factor for this condition [1,2,4].

To the best of our knowledge, this is the first reported case showing complete resolution using topical antibiotics (mupirocin) and antiseptics (bismuth subgallate). Further studies need to be done to confirm the efficacy of topical antibiotics and antiseptics in treating erosive pustular dermatosis. 

## Conclusions

To conclude, erosive pustular dermatosis of the scalp is a rare inflammatory skin disorder that commonly affects elderly patients with sun-damaged skin and androgenic alopecia. It has been associated with multiple autoimmune diseases, and physicians have relied on topical potent corticosteroids as the most effective treatment. This case report describes the first case of erosive pustular dermatosis of the scalp being associated with vitiligo and successfully treated with topical antibiotics and antiseptics.

## References

[1] Starace M, Alessandrini A, Baraldi C, Piraccini BM (2019). Erosive pustular dermatosis of the scalp: challenges and solutions. Clin Cosmet Investig Dermatol.

[2] Bhargava S, Yumeen S, Henebeng E, Kroumpouzos G (2022). Erosive pustular dermatosis: delving into etiopathogenesis and management. Life (Basel).

[3] Molle MF, Burroni AG, Herzum A, Parodi A (2022). Erosive pustular dermatosis of the scalp and multiple sclerosis: just a coincidence?. Dermatol Reports.

[4] Di Bartolomeo L, Ceravolo I, Borgia F, Vaccaro F, Guarneri C, Vaccaro M (2023). Treatment of erosive pustular dermatosis of the scalp: our experience and review of unconventional topical drugs. Eur Rev Med Pharmacol Sci.

[5] Junejo MH, Kentley J, Rajpopat M, Tan XL, Mohd Mustapa MF, Harwood CA (2021). Therapeutic options for erosive pustular dermatosis of the scalp: a systematic review. Br J Dermatol.

[6] Gupta S, Chopra D, Preeyati Preeyati (2023). Erosive pustular dermatosis of the scalp secondary to erlotinib: a rare occurrence with trichoscopic perspective. J R Coll Physicians Edinb.

